# A situational analysis of primary health care centers in Brazil: challenges and opportunities for addressing mental illness and substance use-related stigma

**DOI:** 10.1017/S1463423622000251

**Published:** 2022-07-01

**Authors:** Sireesha Jennifer Bobbili, Bruna Sordi Carrara, Raquel Helena Hernandez Fernandes, Carla Aparecida Arena Ventura

**Affiliations:** 1 PAHO/WHO Collaborating Centre for Addiction and Mental Health, Institute for Mental Health Policy Research, Centre for Addiction and Mental Health, Toronto, ON, Canada; 2 University of São Paulo at Ribeirão Preto College of Nursing, Ribeirão Preto, São Paulo, Brazil; 3 University of São Paulo at Ribeirão Preto College of Nursing, PAHO/WHO Collaborating Centre for Nursing Research Development-Brazil, Department of Psychiatric Nursing and Human Sciences, Vice Coordinator, Unit of the Institute of Advanced Studies – Ribeirão Preto, São Paulo, Brazil

**Keywords:** addiction, equity, mental illness, primary health care, stigma, substance use

## Abstract

**Background:**

The detrimental impact of stigma toward people with mental illness and substance use problems (MISUP) is well documented. However, studies focusing on stigma reduction in Latin American primary health care (PHC) contexts are limited. This situational analysis incorporating a socioecological framework aims to provide a comprehensive understanding of MISUP-related stigma in PHC centers in Brazil. The objectives of this analysis are twofold: (1) to understand the current mental health and substance use service delivery context and (2) identify challenges and opportunities for addressing MISUP-related stigma in PHC centers in Ribeirão Preto, Brazil.

**Methods:**

Environmental scans of four Family Health Units were conducted in early 2018 to explore population needs and service delivery for individuals with MISUP. In addition, a symposium was organized in October 2018 to consult with diverse stakeholders and gather local perspectives about MISUP-related stigma conveyed in PHC settings. NVivo 12 software was used to conduct a thematic analysis of the qualitative data collected from the environmental scans and the symposium consultation.

**Results:**

Themes identified at the national level in the socioecological framework indicate that political support for national policies related to reducing stigma is limited, particularly regarding social inclusion and the decentralization of mental health services. Themes at the regional, organizational, and interpersonal levels include insufficient mental health expertise and the limited involvement of those with lived experience in decision-making. Suggestions for stigma interventions were provided, including increased contact with individuals with lived experience outside of client-patient interactions, capacity building for professionals, and public education campaigns.

**Conclusion:**

Increased government support, capacity building, and promoting social inclusion will provide opportunities to reduce stigma and reach marginalized populations. These findings will assist with addressing current gaps in PHC mental health service provision and may inform anti-stigma strategies for Brazil and other Latin American low- and middle-income countries.

## Introduction

Mental illness and substance use problems (MISUP), such as depression, anxiety, alcohol, and illicit drug abuse, are major public health issues affecting more than 1 billion people worldwide (Global Burden of Disease Study, 2016 as cited in Rehm & Shield, 2019; PAHO, 2019). Brazil possesses certain demographic and economic characteristics that have consistently been associated with the increased incidence and persistence of mental disorders in the general population (GBD, 2016). According to recent estimates, depressive and anxiety disorders were the fifth and sixth causes of years of life lived with disability in Brazil, respectively (GBD, 2016).

This global burden of disease is exacerbated by the mental health treatment gap, where only a small portion of people affected by MISUP receive much needed treatment (Wainberg *et al*., 2017; Evens-Lacko *et al*., 2018). This treatment gap is greater than 50% in all countries but may reach up to 90% in low- and middle-income countries (LMIC), such as Brazil, which is currently identified as an upper middle-income country (Patel *et al*., 2010; World Bank, 2019). A variety of factors contribute to this gap in LMIC, including limited human resources, uncoordinated service delivery, and inadequate policies (Wainberg *et al*., 2017). Stigmatizing attitudes and discriminatory behaviors are also major barriers which prevent people from accessing, receiving, and adhering to treatment, effectively widening the treatment gap (Sapag *et al*., 2018; Ronzani *et al*., 2009).

Stigma is a social process that is defined as “labelling, stereotyping, separating, status loss, and discrimination co-occur[ing] in a power situation” (Link & Phelan, 2001, p.367). MISUP-related stigma has a detrimental impact on individuals by inducing feelings of shame, guilt, hopelessness, anger, and low-self efficacy, which limit help-seeking behaviors (Soares *et al*., 2011). Stigma conveyed by health professionals also contributes to the lived experience of MISUP by negatively influencing the initiation and maintenance of treatment (van Boekel *et al*., 2013).

Research indicates that stigma varies across cultures and contexts (Grandbois & Grandbois, 2005). Given the unique historical, social, cultural, and political contexts in Latin American countries, specifically Brazil, stigma must be studied where it occurs to accurately understand how local populations define and experience it (Grandbois & Grandbois, 2005). Research in local contexts is also necessary for developing effective interventions that address stigma and discrimination in a culturally safe manner, which can ultimately improve the health equity of populations affected by MISUP. This situational analysis using a socioecological framework aims to provide a comprehensive understanding of MISUP-related stigma in PHC centers in Brazil. The objectives of this analysis are twofold: (1) to understand the current mental health and substance use service delivery context and (2) identify challenges and opportunities for addressing MISUP-related stigma in PHC centers in the municipality of Ribeirão Preto, Brazil.

Situational analyses are helpful when developing and implementing research studies in contexts where mental health services and resources are limited (Murphy *et al*., 2019). They are also useful for understanding the current state of mental health services, providing insight into health equity gaps, and identifying opportunities for service delivery improvement (Rasanathan & Diaz, 2016). Incorporating a socioecological framework into this analysis provides additional advantages. By exploring the interaction of the social environment at the national and regional levels with attitudes and behaviors at the organizational and interpersonal levels, a comprehensive view of stigma may be generated (Stokols, 1996; Qin & Song, 2021). For example, the influence of national and regional policies on interactions in organizational settings and at the interpersonal level is important to consider when investigating MISUP-related stigma in PHC settings. By using this approach, findings from this study will be particularly relevant for informing stigma-related research and designing interventions that are ultimately well suited for the Brazilian context.

### Brazil’s mental health care system

The evolution of Brazil’s health system is relevant for understanding the contemporary mental health context. After the fall of Brazil’s military dictatorship, the publicly funded Unified National Health System (SUS) was created in 1988 to encourage equitable services and widespread access to medical care (Onocko-Campos, 2019; Almeida, 2019). Mainly financed by taxes with contributions from federal, state, and municipal budgets, the SUS ensures that publicly financed health services and common medications are universally accessible and free of charge (Macinko & Harris, 2015).

Based on the Caracas Declaration, Brazil’s National Mental Health Policy was later developed and closely aligns with SUS principles by concentrating on decentralizing mental health services from psychiatric hospitals (Onocko-Campos, 2019; Almeida, 2019). With a focus on human rights, the National Mental Health Policy encouraged psychiatric reform in the country, leading to a community-based approach to primary mental health care (Onocko-Campos, 2019). As a result, PHC (also known as basic care in Brazil) provides direct access to public mental health services offered through the SUS.

Centres for Psychosocial Care (CAPS) are replacing psychiatric hospitals and now comprise most community-based mental health services in Brazil (Onocko-Campos, 2019; Almeida, 2019). Considered to be the backbone of the mental health service network, CAPS provide outpatient services for individuals with severe MISUP, including specialized CAPS to serve children and youth and those recently discharged from inpatient treatment services (Almeida, 2019). A limited number of CAPS also provide 24-hour emergency, partial hospitalization, and rehabilitation services (WHO & Brazil Ministry of Health, 2007). Since CAPS are a major component of the community-based mental health network in Brazil, they must work closely with other PHC structures, such as family health teams at Family Health Units (FHUs).

FHUs were chosen as the research setting for this study as they are typically the first point of contact for individuals affected by MISUP in Brazil. Since MISUP-related stigma among PHC professionals is common, an opportunity exists to significantly improve mental health services by developing effective and relevant anti-stigma interventions (Scazufca *et al*., 2016; Santos, Barros & Santos, 2016).

## Methods

### Environmental scans

FHUs in Ribeirão Preto with four family health teams were chosen as the focus for the environmental scans since they are typically faced with a high demand for mental health care. Managers at four randomly selected FHUs with four family health teams in Ribeirão Preto were contacted via telephone in early 2018. An explanation of the study was provided and all four FHUs agreed to participate. The environmental scans aimed to better understand population needs, service delivery, and determine opportunities for implementing anti-stigma interventions.

The environmental scan’s dimensions of analysis included the functioning and structure of FHUs; access, care, and integration of individuals with mental illness at FHUs; challenges for family health teams when dealing with patients with mental illness; and existing mental health public policies and programs. Information was collected from multiple sources, including public domain information, such as annual reports from the Municipality and the Ribeirão Preto Health Department website. In addition, FHU key informants were consulted to fill in any information gaps. Given FHU manager responsibilities include planning, management, organizing work processes, coordinating activities in their catchment areas, and overseeing the integration of the FHUs with other services, the four managers (one from each FHU) who were initially contacted about the study were selected as key informants.

### Stakeholder consultations

A one-day symposium was arranged at the University of São Paulo in Ribeirão Preto in October 2019. The Symposium brought together diverse PHC stakeholders who were interested in sharing local perspectives about MISUP-related stigma conveyed in PHC settings, including challenges and opportunities to improve services. Various key stakeholders from Ribeirão Preto were intentionally recruited. For instance, CAPS health professionals, the coordinator of the Family Health Strategy, a manager from a Basic Health Unit (another type of PHC center), and two members of the Local Health Council and the Municipal Health Council were contacted with information about the symposium. FHU managers who participated in the environmental scans were also invited but were unable to attend. However, they assisted with recruiting FHU service users and their family members. Service users from various types of CAPS and Municipal Health Units were invited by research team members to participate as well. The majority of stakeholders accepted the invitation.

To ensure service users with lived experience and their family members were included throughout the planning process, a meeting was organized to discuss symposium content. A CAPS social worker, three service users, and one family member of a service user participated in the planning meeting. These individuals also agreed to attend the Symposium and participate in certain panel discussions.

In total, 88 key stakeholders from Ribeirão Preto attended the Symposium, entitled “Mental Health, Stigma and Discrimination: Experiences, Challenges and Lessons Learned in Primary Care.” The Symposium began with two presentations regarding stigma research in Brazil and Canada, followed by two roundtable discussions. The first discussion focused on MISUP-related stigma and equity issues and the second on stigma and the integration of mental health and substance use care into PHC. The final portion of the symposium involved three working groups which discussed the potential for a stigma reduction intervention specifically designed for FHUs.

Research team members facilitated the various Symposium components and guided the discussions with prepared questions. The consultations were conducted in Brazilian Portuguese by the Brazilian authors. Handwritten notes were taken in Portuguese and later translated into English by Brazilian research team members who are fluent in both languages.

This methodology integrates both qualitative data and secondary data from the FHU environmental scans and symposium consultations with stakeholders (Murphy *et al*., 2019). Qualitative data were coded by the Canadian member of the research team using a thematic analysis approach guided by the socioecological framework. Qualitative data analysis software, NVivo 12, was used for the analysis, and findings were triangulated between the various data sources where possible.

## Results

### National level: legislation and policies for mental health and substance use in primary care

#### Brazil’s National Policy for Primary Care

The National Policy for Primary Care focuses on health promotion and protection, illness prevention, diagnosis, treatment, rehabilitation, harm reduction, palliative care, and surveillance (Almeida *et al*., 2018). This policy stipulates that primary care should be administered through integrated practices and delivered by qualified multiprofessional teams for a defined geographic area (Couttolenc & Dmytraczenko, 2013).

#### Brazil’s Family Health Strategy

The National Policy for Primary Care prioritizes the Family Health Strategy (FHS) for the expansion, delivery, and quality of primary care (Couttolenc & Dmytraczenko, 2013; Almeida *et al*., 2018). Developed in 1994, the FHS was introduced to provide integrated primary care using multidisciplinary health professional teams (Couttolenc & Dmytraczenko, 2013). It also increased accessibility by placing health professional teams close to the community, enabling the delivery of consistent and continuous care to the most vulnerable and marginalized (Paim *et al*., 2011; Macinko & Harris, 2015; Almeida *et al*., 2018).

The FHS provides services through PHC centers called Family Health Units (FHUs). Each FHU offers services via one or more Family Health Team (FHT), which have been fundamental in reducing accessibility related inequities (Almeida *et al*., 2018). However, due to variations in populations and local resources, the FHS continues to require adaptation to ensure it adequately addresses local needs.

In addition to family registration, health promotion, and disease prevention responsibilities, FHTs are expected to promote collaboration between organizations to increase positive citizenship. Multiple FHTs housed within each FHU are responsible for monitoring health status in their catchment area via home visits and case discussion meetings, staff meetings to address FHU issues, and updating Ministry of Health databases (Almeida *et al*., 2018; Andrade *et al*., 2018).

The FHS encourages a high level of cooperation among professionals in each FHT. Therefore, planning is a shared responsibility and decisions must be agreed upon by team members. Each FHT includes a physician, nurse, nursing assistant, and four to six community health workers at minimum (Almeida *et al*., 2018; Andrade *et al*., 2018). Community health workers are expected to live in the catchment area, regularly visit households, and develop rapport with community members (Soares & Oliveira, 2016). FHTs may also include other health professionals depending on population needs (Soares & Oliveira, 2016).

FHTs are organized geographically, covering populations of up to 1000 households or 4000 people, with no gaps between catchment areas (Andrade *et al*., 2018). Each team member has defined roles and responsibilities, with national guidelines directing FHS responses to most health problems. The FHS has been rapidly scaled-up across the country, increasing from 2,000 FHTs covering 7 million people (4% of the Brazilian population) in 1998 to 39,000 teams serving 123 million people (63% of the population) in 2015 (Andrade *et al*., 2018).

#### Integration of mental health & substance use services into primary care

The FHS guides primary care reorganization according to the Unified Health System (SUS) and favors the social inclusion of individuals affected by MISUP by ensuring the availability of local mental health services (Mateus *et al*., 2008). This reform has become essential for people with MISUP and their families, especially since the Ministry of Health encourages the Brazilian population to use local rather than centralized mental health services.

Both the National Policy for Primary Care and the FHS recommend decentralization and local availability of PHC services; however, stakeholders indicate that mental health services are inadequately integrated into PHC and patient needs are typically not considered:

*“The integration between mental health and primary care is fundamental, but I feel very stuck. The system does not give much opportunity to provide care in the way we would like to. I need, as unit manager, to comply with protocols and often care is not as it should be. It is missing the space to ask the patient: What is your need?” (Health Unit Manager)*

In addition, capacity building for health professionals is not supported by existing policies. Therefore, many professionals do not possess the necessary skills or expertise to provide adequate mental health care. One Local Health Council member stated:

*“It is very common that many health professionals who work in this area of mental health are not able to do so, because [there is a] lack of policy for training…training is not understood as a course and training needs to actually go beyond a course” (Municipal Health Council Member).*

Without opportunities for training, health professionals are inadequately equipped to provide effective care and focus on complying with protocols rather than humanizing treatment for patients.

#### The Matrix approach and Family Health Support Nuclei

The Matrix approach supports the new community-based mental health model by urging health professionals to understand local employment, education, social services, and cultural resources so they can offer mental health supports that go beyond clinical care (Mateus *et al*., 2008). For the Matrix approach to work effectively, health professionals must receive professional training to offer services in this manner (Soares & Oliveira, 2016).

Family Health Support Nuclei assist general FHTs by providing specialized care. For instance, psychologists or psychiatrists are central members of Support Nuclei that address mental health issues (Soares & Oliveira, 2016). However, municipal health managers ultimately decide the composition of each Support Nuclei based on local needs (Soares & Oliveira, 2016).

Both strategies are somewhat new and complex and are therefore difficult to implement. Professionals must understand their roles as a part of larger teams as per the FHS to be effective:

*“It is best for the team to better understand the team as a whole. This reorganization is happening in an intense and even heavy way. We are suffering some criticism. The changes are not easy.” (Family Health Strategy Coordinator)*

Rapport and trust must be developed among multidisciplinary team members, regardless of differing theoretical frameworks which underlie various professions (Soares & Oliveira, 2016):

*“Matrix support as an initial action is being implemented in some services. Developing these spaces is not a simple task. By entering communities, you will deal with beliefs, with values, thoughts, fears. And until you say, look, I’m here with you, it’s something that’s difficult to build.” (Social Worker)*

Although multidisciplinary teams with a range of expertise are considered to be a strength of the Matrix approach, problems still persist within mental health care:

*“The professionals thought someone had arrived who will save our homeland: the specialized professional. More problems were raised, rather than seeking solutions. The articulation of the network is a very slow process. There are many, many problems in the [mental health] specialty.” (Social Worker)*

Stakeholders highlighted certain realities regarding recovery and treatment opportunities. One professional indicated that FHUs have the potential to address issues before they become chronic if professionals are skilled in harm reduction and appropriate structures are in place to support this perspective:

“…*But when we began the conversations, explaining that these spaces are for care, there was a greater understanding and [our] speech began to change. For instance, service users dealing with anxiety may not be cured but may receive care that helps with symptom control, an individual who uses alcohol and drugs will sometimes have relapses, the person who has a psychiatric disorder…will have a crisis at some point. There is often a lack of structures to accommodate these patients before the situation becomes critical… this is a problem of every service. We have begun to understand that this space is for building citizenship and quality of life.” (Social Worker)*

Stakeholders stressed the Matrix approach must be supported by everyone involved to be effective:

*“Support for the Matrix approach is necessary. It needs political commitment and management, from the secretariat, from the health units, from the management of the units. If care is not shared, it cannot be provided. The team must be committed to Matrix support.” (Family Health Strategy Coordinator)*

### Regional level: the current landscape of Ribeirão Preto

#### Division of mental health and substance use care between primary care and specialized services

To meet the demand for health care, the municipality of Ribeirão Preto recommends that each FHT serve a maximum of 3,500 people, with at least one community health agent who serves no more than 650 people. Ribeirão Preto currently has 45 FHTs; however, Ordinance No. 1.737 (July 12, 2017) has increased the allowable number of FHTs to 80 in the municipality.

Although policies have identified organizational responsibilities, specialized services seem to discourage patients from returning to PHC (i.e., basic care centers) for general health services, as there is often confusion regarding the division of care:

*“Some patients are already being accommodated by these [Family Health] teams and we have difficulties, when many times, CAPS, ambulatories, they take care of that patient and the patient does not “return” to Basic Care. It’s as if the patient were ours, so we take over, sometimes because the professional who is there does not have the training to follow, but also because they think it is their obligation.” (Family Health Strategy Coordinator)*

One stakeholder identified an innovative strategy for tracking patients with MISUP while they access specialized services and believe that PHC should oversee all care to ensure appropriate and continuous support is provided:

*“This patient lives in one area and we are able to follow [them] through their family members, so that patient is not alone in an outpatient or specialized mental health service. Around them they have a family and behind the family, there is a community where he lives. And that family doctor, at that moment, should be better able to have that integral look.” (Family Health Strategy Coordinator)*

In some cases, mental health and substance use services in PHC are being used for emergency care, rather than decentralized mental health care at the local level. Therefore, health professionals are overburdened and unable to provide adequate supports to those with MISUP, resulting in the overdiagnosis of problems:

*“How do I see that health professionals are dealing with patients with mental disorders? The same way [they deal with] inequities. Particularly the doctors, but also nursing, we see a departure from care for the population. We turn anyone into an outpatient. The CAPS are outpatient clinics, the Primary Care Center turns into a large ambulatory and I go there to do what? Treatment of something urgent, so I cannot provide CARE for the person I need to take care of, so we end up medicalizing everything.” (Municipal Health Council Member)*

#### Civil society participation through health councils

When Brazil’s publicly funded health system, the SUS, was restructured, Health Councils were created to promote civil society participation in the health sector (Martinez & Kohler, 2016). Developed in 1990, Health Councils were established to advise, monitor, and hold governments accountable for implementing health policies at all levels (Martinez & Kohler, 2016). They were also intended to deter corruption and provide a forum for civil society participation in decision-making processes for health system issues.

Health Councils are comprised of 48 members in total, half of whom are service users, a quarter are healthcare representatives, and the remaining quarter are government officials (Martinez & Kohler, 2016). While service users are integral to Health Councils, they continue to be excluded from discussions about mental health care:

*“We have some questions to be discussed: adjust CAPS (Psychosocial Care Centers), adapt Therapeutic Residences, expand the support Matrix and expand community groups. We have seen the expansion of some discussions in mental health, especially when it comes to depression and suicide, but it does not widen to include users and network workers.” (Municipal Health Council Member)*

Health Councils are highly regarded throughout Brazil; however, there seems to be a disconnect between federal policies and local realities. While the FHS prioritizes mental health integration into PHC, this vision has not been prioritized on a long-term basis in Ribeirão Preto:

*“In 2015, between March and September, the council created a Mental Health Commission… It began, and mainly questioned the work of Therapeutic Communities. We did not see a network structure according to what the Ministry foresaw…[they] had some very interesting proposals and treated mental health care in a very broad way…it lasted 6 months. Although it had broader objectives, members began to discuss the issue of coverage, the question of services, and saw that the information was not only for management…and it’s over. At the time, the mental health coordinator did not want to [engage in] some discussions, so the Commission itself decided not to exist anymore.” (Municipal Health Council Member)*

### Organizational level: the primary health care system

#### Mental health and substance use treatment challenges in primary health care

Although many health professionals have received mental health training, some avoid discussing inequities faced by patients with MISUP by focusing exclusively on treatment:

*“We have a context of health inequities that are not beneficial to our mental health…How should we address these health inequities? Maybe this is the hardest, it is not exclusive to mental health. They are present and no one wants to discuss them. We want to treat, medicalize, and recommend the patient for a psychological follow-up, instead of questioning the social construction that produces these mental illnesses or [things that] cause mental health problems.” (Municipal Health Council Member)*

According to one social worker, service users do not necessarily perceive health professionals in a positive manner, which can be challenging when providing care:

*“How do we approach each other’s daily lives? It’s not easy. How do we access the space that often we are not seen as someone who will help?” (Social Worker)*

#### Lack of collaboration and familiarity with evidence-based recovery models

The treatment approach taken by health professionals is not always discussed among team members due to differing theoretical frameworks, unfamiliarity with evidence-based recovery models and limited knowledge about the roles and responsibilities of various professions. Consultation with service users regarding their treatment plan may also be omitted, leading to mistrust. For example, harm reduction is an important strategy that supports individuals who struggle with substance misuse; however, abstinence may also provide a viable option for some. Therefore, health professionals must be knowledgeable and willing to collaborate with team members as well as service users to discuss treatment options:

*“Treating the user of alcohol and drugs without discussing abstinence is impossible. Reducing damage, even today, despite having the advances [frightens] a lot of people. But when you start working with professionals, with society, with the community, things can change.” (Social Worker)*

Another challenge is the prescription or over prescription of medication without an appropriate explanation. This can lead to difficulties for service users, especially when their daily functioning is affected:

*“Haldol; take sleeping pills. You wake up and take medicine to sleep. And then after a while you leave. So, what happens? You don’t recognize yourself in the world. You don’t know who you are. You don’t know what things mean because things had so much meaning before, and now they have no meaning. And then it doesn’t make much sense to take care of yourself.” (Health Service User)*

Once treatment or medication is provided, follow-up is necessary to determine impacts or adjust treatment. One service user described the lack of treatment monitoring and evaluation and how this can lead to institutionalization:

*“We don’t measure the consequences very much…and this is a very complicated thing because we end up getting into a lot of things. And then we arrive in a state that [is] no way to live…We acquire a pattern of behavior that is totally unacceptable in the society in which we live, and we end up being picked up by some institution.” (Health Service User)*

### Interpersonal level: interaction between health professionals and services users with mental illness and substance use issues

#### Labelling

Participants identified negative terms used by health professionals to label individuals with MISUP, such as “crazy,” “noodle,” and “tramp”. These labels imply character flaws or blame individuals for their current mental state, leading to stigma and discrimination. One service user also indicated that medical terms do not accurately capture the experience of people with MISUP, and it is important to understand diagnoses from the perspective of service users:

*“I am a mental health service user…I am diagnosed with bipolarity. The term used in the medical field does not represent all that bipolarity is. In fact, the experience is very extreme and it is important to share this from the perspective of those who live [with it].” (Health Service User)*

#### Lack of involvement of people with lived experience

The typical patient–provider relationship is characterized by a power imbalance, with the health professional positioned as the expert, directing the patient on a specific course of treatment. Without proper training, health professionals may not recognize that people with MISUP can provide valuable input that may be helpful for their treatment and recovery. One social worker emphasized the need to humanize care and develop new strategies to collaborate with patients, otherwise recovery may be jeopardized:

*“We need to help the stigmatized person…to have a new story to tell and we have to believe this story as well. It is very important that we, as a society, but as health professionals in health care contexts, build new ways of living with the people who are there for our care. These incoming people are not just users of substances or have some mental disorder, they are human beings who have a name, identity and who have much to contribute…[otherwise]they stay in [their current] condition.” (Social Worker)*

### Recommendations for addressing stigma in Brazil

#### Improve health literacy

Stakeholders expressed that the general population is unclear about their rights, the laws that govern mental health care and the function of health care structures, particularly which institutions provide mental health versus general services. Health professionals also possess limited knowledge about the mental health system and division of care across agencies, indicating that this group should be included in health literacy promotion.

#### Increase collaboration within multidisciplinary teams

Given the important role of multidisciplinary health teams in PHC, suggestions included strengthening team dynamics to improve mental health care. For example, stakeholders felt that developing camaraderie will result in improved communication among team members and encourage discussion about common practices, such as effective approaches for treating individuals with MISUP in non-stigmatizing ways. This may also provide opportunities to discuss how to best improve practice:

*“Dealing with stigma can force us out of our comfort zone. It is important that the health professional has a profile to work in these spaces. We need to start working with teams on “how am I doing?”; “How can I work in a good way for the other and for myself?” (Social Worker)*

#### Increase inclusion of people with lived experience

From a policy perspective, stakeholders indicated that health services should better support social inclusion policies as part of the Matrix approach. For instance, identifying roles that health professionals can play to promote social inclusion while carrying out their regular duties. Including people with MISUP in all aspects of society, not just decisions about mental health treatment and care, was also suggested. Participants emphasized the need for active social participation that builds on current structures:

*“The deconstruction of stigma is related to the construction of a new society…we need to get out of our services, get out of our institutions and get closer to the population; search for alternative spaces. Revitalize this basic process, together with the neighborhoods, with the local councils…to create a space that consolidates all others. It is difficult to [participate collectively] without active social participation.” (Municipal Health Council Member)*

#### Increase investment in training for health professionals

Positive patient–provider interactions are necessary for delivering non-stigmatizing mental health care. Stakeholders stressed the importance of government investment in mental health training. By learning about MISUP and associated stigma and discrimination, participants suggested that health professionals will become aware of their influence on help-seeking behaviors and treatment adherence, thus improving interpersonal and communication skills. Stakeholders also felt that training which incorporates reflection about personal biases would lead to increased sensitivity from all health professionals, not just mental health specialists, and inspire non-stigmatizing treatment. One participant posed important questions to be considered by all professionals providing mental health services:

*“The goal is to return to the question of how to fight health inequities. How can we unite beyond a pathology? How do we build a social process that goes beyond diminishing these inequities? How can we welcome this population and welcome us?” (Municipal Health Council Member)*

#### Opportunities for contact-based education

Contact-based education was deemed important and necessary for increasing opportunities for interaction between health professionals and those with MISUP outside of the patient–provider relationship. Participants indicated that organized events, such as the Symposium, are helpful for bringing diverse individuals together to exchange ideas and encourage conversations with people with MISUP, who are typically excluded from society. Trainings that allow health professionals, service users, and family members to connect virtually were also suggested.

## Discussion

This situational analysis using a socioecological framework explores the current state of MISUP services in Brazil, which highlights strengths, challenges, and opportunities for addressing associated stigma. From a socioecological perspective, a unique set of problems exist at various levels, all of which converge to impact individual experiences of MISUP and reinforce stigma and discrimination in Brazilian PHC settings. Participants recommended various strategies to address stigma, all of which are applicable to the identified problems. Main strengths, challenges, and opportunities are highlighted in Table 1.

**Table 1.** Strengths, challenges and Opportunities for addressing stigma related to mental illness and substance use in PHC in Brazil

Strengths	Challenges	Opportunities
**National Level**		
Federal policies promote decentralization via a community-based model of mental health care to increase access	Mental health services are inadequately integrated into primary care	Renew political commitment and increase funding to mental health service integration and ongoing mental health capacity building for professionals
	Policies do not support capacity building; primary health care workforce does not possess essential mental health skills	
**Regional Level**		
General mental health services are available through PHC organizations and specialized services for severe mental illness and substance use are available through Centres for Psychosocial Care (CAPS)	Confusion regarding the division of care between primary care and specialized services	Improve the health literacy of the general population, service users, family members, and health professionals through health promotion/education campaigns
Civil society participation of service users is promoted through Municipal Health Councils (i.e., Council seats are reserved for service users)	Service users are consistently excluded from discussions about mental health service provision	Promote genuine and meaningful inclusion of service users in Municipal Health Councils; increase social inclusion opportunities in all aspects of society, not just health care
**Organizational Level**		
Multidisciplinary teams include a range of expertise which can address the unique mental health needs in specific geographic areas	Collaboration among team members is difficult due to differing theoretical frameworks and lack of knowledge about roles and responsibilities of various professions Lack of knowledge regarding evidence-based recovery models for mental illness (e.g., symptom control versus return to premorbid state) and substance use (e.g., harm reduction versus abstinence)	Increase investment in mental health training for health professionals that develops mental health knowledge and treatment expertise, and promotes collaborative approaches to care
**Interpersonal Level**		
Primary health care professionals are typically the first point of contact for individuals affected by mental illness and substance use which provides an opportunity to offer sensitive, empathetic care	Patients with lived experience may be labeled in a negative manner by health professionals and are excluded from treatment decisions	Increase investment in training for health professionals involving contact-based education; promote shared decision-making for treatment decisions

At the national level, Brazil’s federal policies promote decentralization to improve access to mental health care. However, our results indicate that mental health services continue to be ineffectively integrated. Many Latin American countries have been working toward decentralization with varying degrees of success (Minoletti *et al*., 2012). Globally, mental health system reform has been slow, mainly due to limited political will and inadequate funding (Almeida *et al*., 2018; Minoletti *et al*., 2012). Interestingly, Minoletti and colleagues (2012) found that Brazil is one of three Latin American countries that have successfully changed from a hospital-based mental health system to a community-based model, even though our study results indicate that more work is necessary to become fully decentralized. Our results also suggest that the PHC workforce does not possess essential mental health skills and expertise to address population needs.

In terms of opportunities, stakeholders did not directly raise renewing political commitment and increasing funding, as they may have assumed this to be an obvious issue since mental health funding has decreased over time (Minoletti *et al*., 2012). The 2014 economic crisis compromised the financial situation in Brazil, resulting in federal fiscal austerity policies and limited resources for the SUS (Vieira & Benevides, 2016; Funcia & Ocke-Reis, 2018; Vieira *et al*., 2019). Redirecting funding to mental health decentralization and capacity building would allow for more effective interventions, shorter wait times, greater continuity of care, and increased satisfaction with services (Beaulieu *et al*., 2017).

At the regional level, general and specialized mental health care is available through PHC organizations, such as FHUs and CAPS, respectively. Confusion regarding the division of care is persistent, particularly with respect to the provision of PHC services and emergency mental health services, which can lead to increased burden on health professionals who are ill-equipped to provide appropriate care. Stakeholders suggested improving the health literacy of the general population, service users, their family members, as well as health professionals, which would address confusion regarding the division of care between PHC and specialized mental health services and alleviate the strain on certain institutions.

Another regional level challenge is genuine civil society participation by people with lived experience. Although Municipal Health Councils reserve seats for service users, our results show that those with MISUP are consistently excluded from service provision discussions, an area where their perspectives would improve treatment. Brazil has enacted progressive social inclusion policies that promote a more equitable society for people with MISUP. However, excluding service users, particularly Heath Council members, from discussions about mental health care can hinder service improvement and cause further stigmatization.

At the organizational level, innovative multidisciplinary teams as part of the mental health Matrix approach can address the unique needs in catchment areas. Yet, our results indicate that teams are functioning suboptimally due to differing theoretical frameworks among professionals and the unclear division of labor. Results also suggest government-supported capacity building initiatives are nonexistent, resulting in inadequate MISUP treatment knowledge. This finding is supported by Gerbaldo and colleagues (2018), who surveyed 29,778 FHTs across Brazil and found that 60.3% of participants felt unprepared to work in the mental health field (Gerbaldo *et al*., 2018).

An inadequately skilled workforce can contribute to misconceptions about MISUP. Non-specialist PHC professionals who provide mental health care may be reluctant to do so without proper training, thereby decreasing access and acceptance of those affected by MISUP (Kakuma *et al*., 2011). This can negatively affect the quality of care by perpetuating social distancing and stigmatizing attitudes in institutional spaces (Nunes & Torrente, 2009; Ng, Rashid & O’Brien, 2017).

While evidence shows that capacity-building initiatives can improve attitudes and strengthen existing human resources, it has failed to attract sufficient government support (Villani & Masfety, 2017; Ng *et al*., 2017; Tawiah *et al*., 2015; Kakuma *et al*., 2011; Wainberg *et al*., 2017). Intervention studies show that health professional training initiatives incorporating contact-based education are successful at reducing stigmatizing attitudes and behaviors (Link *et al*., 1989; Thornicroft *et al*., 2009; Corrigan *et al*., 2012; Griffiths *et al*., 2014; Stubbs, 2014; Ng *et al*., 2017). Mentorship and leadership programs are also potential options for building the capacity of a mental health workforce and improving interpersonal skills, practices, and attitudes toward MISUP (Kakuma *et al*., 2011).

At the interpersonal level, our results indicate that health professionals treat patients with MISUP in a paternalistic manner and typically exclude them from treatment plan development. As well, patients are frequently labeled with negative terms, leading to stigmatizing ideas about MISUP among health professionals and the general population. Shared decision making is one strategy that can correct power imbalances between patients and providers (Del Piccolo & Goss, 2012; Health Foundation, 2012; Sapag *et al*., 2018). Collaboration can encourage positive patient–provider relationships involving empathy and respect, thus improving treatment adherence and reducing stigma (Hamann *et al*., 2008; Sapag *et al*., 2018).

This study engaged with a variety of stakeholders whose first language is Brazilian Portuguese. Notes were taken in Portuguese and later translated into English to allow the Canadian researcher to analyze the data alongside the Brazilian research team. As a result of the English translation and analysis, we recognize that there may be some loss of meaning or misunderstanding of certain terms. However, the Brazilian researchers ensured that selected quotes have not been taken out of context.

## Conclusion

Our situational analysis revealed a comprehensive perspective of the mental health service delivery context and the various factors that influence the experience of mental health and MISUP-related stigma in Brazilian PHC settings. While all identified challenges may not be unique to Brazil, contextual factors at national, regional, organizational, and interpersonal levels must be considered when developing anti-stigma strategies (Thara & Srinivasan, 2000). Within a country with a unique history of decentralization and a complex model of community-based mental health care, it is important to devise comprehensive, multi-pronged anti-stigma strategies that build on existing strengths (Shim & Rust, 2013) and take advantage of opportunities to renew political commitments, improve health literacy, increase capacity building, and promote collaborative approaches to treatment. Moreover, involving stakeholders from multiple levels and sectors and well as including people with lived experience is necessary to ensure that MISUP-related stigma is adequately addressed. These findings will assist with addressing current gaps in PHC mental service provision and will inform anti-stigma strategies in PHC for Brazil and other low- and middle-income Latin American countries.
